# Mutual Influences between Nitric Oxide and Paraoxonase 1

**DOI:** 10.3390/antiox8120619

**Published:** 2019-12-05

**Authors:** Marta Marín, Carlos Moya, Salvador Máñez

**Affiliations:** 1 Departamento de Farmacia, Facultad de Ciencias de la Salud, Universidad Cardenal Herrera-CEU, CEU Universities, C/ Ramón y Cajal s/n, Alfara del Patriarca, 46115 Valencia, Spain; marta.marin1@uchceu.es; 2 Departament de Farmacologia, Facultat de Farmàcia, Universitat de València, Av. Vicent Andrés Estellés s/n, Burjassot, 46100 València, Spain; carmoa@alumni.uv.es

**Keywords:** antioxidant, nitric oxide, paraoxonase, reactive oxygen species, serum lipoprotein, vascular inflammation

## Abstract

One of the best consolidated paradigms in vascular pharmacology is that an uncontrolled excess of oxidizing chemical species causes tissue damage and loss of function in the endothelial and subendothelial layers. The fact that high-density lipoproteins play an important role in preventing such an imbalance is integrated into that concept, for which the expression and activity of paraoxonases is certainly crucial. The term paraoxonase (aryldialkyl phosphatase, EC 3.1.8.1) encompasses at least three distinct isoforms, with a wide variation in substrate affinity, cell and fluid localization, and biased expression of polymorphism. The purpose of this review is to determine the interactions that paraoxonase 1 has with nitric oxide synthase, its reaction product, nitric oxide (nitrogen monoxide, NO), and its derived reactive species generated in an oxidative medium, with a special focus on its pathological implications.

## 1. Introduction

After having overcome some reasonable initial skepticism, the discovery of nitric oxide (NO) as a powerful mediator of vasodilatation opened a very fruitful period in the history of both physiology and pharmacology. It was not only the gaseous character of NO that sustained its conceptual attraction, but also its local origin, its short life, and its peculiar form of synthesis by nitric oxide synthase (NOS) [1]. It is around the last of these characteristics that a wide field of research has developed over the past three decades. The efficacy of the entire NO system was connected with the ambivalent role of cholesterol-carrying plasma lipoproteins due to the well-documented effect of high density lipoproteins (HDL) increasing the activity of endothelial NOS (eNOS) [2]. The third branch of this formal construction consists of the study of paraoxonases (PON), a set of esterase enzymes with low substrate selectivity that is related to antioxidant defense and cardiovascular health [3]. Among these, paraoxonase 1 (PON1) is perhaps the most relevant, due to its abundance and various extracellular effects, in part due to its inclusion in HDL. Although it seems that the presence of PON1 in HDL participates in giving these particles the ability to activate eNOS, things are far from being so simple: eNOS has, in response to different stimuli, an innate instability that leads to what is known as uncoupled eNOS, a form that loses the ability to synthesize NO and acquires the ability to produce significant amounts of superoxide anion [4]. Furthermore, HDL also has a double-sided character, since not all types of HDL are equally good at stimulating eNOS activity. In fact, HDL may become dysfunctional under oxidative stress and, since that induce more oxidative stress, HDL are mediators of a circular harmful pathway. It has been repeatedly shown that alterations in the PON1 molecule not only affect its activity, but they can also contribute to making HDL dysfunctional and worsening their a priori favorable cardiovascular role [5].

On the basis of the aforementioned findings and taking into account that the production of NO, which is initially hypotensive and atheroprotective, can revert to an absolutely opposite state when oxidized to peroxynitrite or nitrogen dioxide, the purpose of the present review is to discuss the recent advances on the causal relationships or, at least, the verified associations between NO and PON1 activity. The analysis is focused to the implication in clinically relevant situations, which are essentially inflammatory or metabolic, but may be of interest even in psychiatric diseases.

## 2. On the Genesis of NO

Nitric oxide (NO) is an important pro-inflammatory cytotoxic agent that protects the host against various pathogens by inactivating and destroying infectious agents [6,7,8]. Under physiological conditions, the gaseous molecule is an important signaling molecule involved in neurotransmission and in the control of vascular tone [9]. NO is produced from L-arginine via the action of NOS in the presence of oxygen, NADPH, and tetrahydrobiopterin. Besides eNOS, two other isoforms have been identified: neuronal NOS (nNOS) and inducible NOS (iNOS) [10]. The large amount of NO produced by iNOS after induction by cytokines or other external stimuli are responsible for the fall in blood pressure in septic shock [11]. They may also contribute to vascular peroxynitrite formation [12] and have been shown to be proatherogenic [13]. In contrast, nNOS and eNOS isoforms are prominently expressed in neurons and epithelial cells, respectively, and both account for constitutively formed low concentrations of NO under physiological conditions. Particularly, eNOS-derived NO has a protective role in the vasculature as eNOS is the predominant isoform in blood vessels. Endothelial NO diffuses to vascular smooth muscle cells where it activates cytosolic guanylyl cyclase, therefore increasing cyclic GMP production and leading to vascular smooth muscle relaxation. It is not only a potent endogenous vasodilator, but also inhibits vascular smooth muscle cell migration and proliferation, platelet adhesion and aggregation, low density lipoproteins (LDL) oxidation, and vascular inflammation [13,14].

To generate NO efficiently, human eNOS must be phosphorylated at its stimulatory sites (Ser615, Ser633, Ser1177, and Tyr81) and dephosphorylated at its inhibitory sites (Thr495 and Ser114) [10]. Protein kinase B (Akt) or AMP-activated protein kinase (AMPK) have been previously identified as the kinases responsible for the classical HDL-induced eNOS phosphorylation and activation at Ser1177 [15]. The non-classical regulation of eNOS activity is based on the formation of redox-active species that trigger adverse phosphorylation by redox-active kinases. Particularly, phosphorylation at Thr495 is mediated by protein kinase C (PKC), which leads to superoxide production and enzyme uncoupling [16]. Endothelial PKCβ has been reported to negatively impact endothelial function by inhibiting stimulatory Akt and eNOS phosphorylation events and enhancing the inhibitory phosphorylation of eNOS at Thr495 [17].

## 3. PON1 as an Antioxidant Component of the Structure of HDL

Structurally, the HDL particle comprises a shell formed by apolipoproteins, phospholipids, and cholesterol surrounding a lipid core containing triglycerides, cholesterol, and its fatty acid esters. The principal *apo* components of HDL are ApoA-I and ApoA-II [18]. ApoA-I expresses its anti-atherogenic properties by transporting cholesterol from the tissues to the liver. High values of the HDL-cholesterol/ApoA-I ratio have been associated with increased cardiovascular mortality and mortality by all causes [19]. PON1, the human serum of PON, is an HDL-associated esterase that binds to HDL by an interaction with ApoA-I and phospholipids [20]. As an esterase, PON1 can hydrolyze lactones, phosphate esters, and lipid peroxide derivatives, and its expression and activity may be modified by certain drugs [21]. According to detailed kinetic studies concerning the main parameters of enzymatic activity (Michaelis constant and catalytic constant) and the features shared with the other isoforms of PON, it was found that the genuine biological function of paraoxonase is to act as a lactonase [22].

Myeloperoxidase (MPO), an important piece of the leukocyte-derived oxidative equipment, causes specific chemical alterations that reduces the beneficial effects of PON1 and ApoA-I. However, PON1 itself is able to inhibit the activity of MPO on the surface of HDL particles, thus decreasing the promotion of lipid peroxidation and other cell-damaging processes. The mechanism of this three-part interaction, soundly explained by Huang et al., begins with the oxidation the Tyr71 residue of PON1 by means of the activity of MPO; then, such residues bind specifically with the P1 an P2 regions within HDL, which is a key step for the activation of PON1. The reciprocal interplay of PON1 and MPO within this ternary protein complex expresses what the authors termed “a fine tuning” of the function of HDL [23]. A few years later, members of the same group established the positions of the PON1 peptidic chain involved in the docking on the surface of HDL. In order to discover this information, Gu et al. described a new protocol based on the use of a synthetic photoactivable diazirinyl phospholipid susceptible to linking with determined amino acid sequences of human PON1 or ApoA1. The residues of PON1 implied in the fixation to HDL were found to be Leu9, Tyr185, and Tyr293, which are quite close and localized in the hydrophobic binding surface [24].

It was reported that PON1 acts as an antioxidant, not only by preventing oxidized LDL (oxLDL) formation, but also by removing the ox-LDL-associated lipids [25]. Accordingly, some studies have recently described that the susceptibility to lipid peroxidation was higher in the HDL isolated from subjects with low PON1 activity than in subjects with higher PON1 activity [26]. On the other hand, the amount of serum amyloid A, an HDL-associated acute-phase protein, increases in the course of inflammation, whereas both ApoA-I and PON1 decrease [27].

Besler et al. [28] evaluated the potential role of malondialdehyde (MDA), an end product of lipid peroxidation, in the effects of HDL on endothelial NO production. They found that although PON1 abundance is nearly doubled in dysfunctional HDL compared to normal HDL, its activity is markedly decreased in the former, potentially inducing a greater formation of MDA. MDA-bound HDL can activate endothelial LDL receptor 1 (LOX-1) and PKCβII, leading to an inhibitory phosphorylation at Thr495 of eNOS and thus decreasing endothelial NO production [29]. Consistent with these findings, some studies demonstrated that MDA content, as well as protein-bound MDA content of LDL and HDL, is a reliable marker of endothelial dysfunction in subjects with low to moderate risk of cardiovascular disease [30].

## 4. Interplay between HDL and NOS

### 4.1. Mechanisms

HDL increases eNOS activity, as has been demonstrated in both in vitro and in vivo studies, but also in humans after applying intravenous reconstituted HDL infusion [29]. NO synthesis induced by HDL partially contributes to HDL’s anti-inflammatory properties by inhibiting the adhesion of polymorphonuclear neutrophils (PMNs) and platelets to the endothelium [31]. In addition, the HDL-induced increase in NO production may be critical to the atheroprotective features of HDL, since NO deficiency is frequently associated with the early progress of hypercholesterolemia-induced vascular disease and atherosclerosis [32]. Further, it was observed that HDL with reduced PON1 content show a compromised ability to activate eNOS in an inflammatory environment, and partially loose the protective effects against cardiovascular disorders [33,34]. The stimulation of eNOS activity by HDL in endothelial cells follows through ApoA-I binding to a high-affinity HDL receptor, scavenger receptor type I (SR-BI), and to sphingosine1-phosphate receptors, which in turn activate Src kinases, phosphatidylinositol 3-kinase (PI3K), and Akt, inducing the eNOS phosphorylation at Ser1177 [18,35]. Seetharam et al. reported that HDL and SR-BI stimulate endothelial cell migration in vitro with potency equivalent to vascular endothelial growth factor, and that the phospholipid, ApoA-I, and cholesterol components of HDL are enough to initiate this cellular response. Nevertheless, in contrast to the mechanisms of action of multiple known stimuli, the promotion of endothelial cell migration by HDL in the study was NO-independent [36].

The formed oxLDL down-regulates NO and contributes to the vascular dysfunction by different pathways. Firstly, oxLDL can lead to the formation of peroxynitrite through the release of oxygen free radicals, decreasing the concentration of NO and affecting the activity of NOS [25]. Secondly, oxidized lipids recognize and activate the endothelial multiligand receptor known as lectin-type oxidized LOX-1 [17], which is considered the main oxLDL receptor in endothelial cells, macrophages, and smooth muscle cells implicated in the pathogenesis of atherosclerosis. LOX-1 is responsible for the binding, internalization, and degradation of oxLDL in endothelial cells by activation of PKCβII and c-Jun N-terminal kinases [37]. Moreover, LOX-1 contributes to the NO dependent endothelial impairment of coronary arterioles by stimulating additional reactive oxygen species (ROS) production and generating a positive feedback loop for further LDL oxidization [38,39,40].

The production of NO by the competent cells may result in an increase of pro-oxidant reactive nitrogen species, which are able to counteract the beneficial effects of PON1 in terms of antioxidant defense. This is not only because of the aforementioned formation of peroxynitrite in the presence of a burst of superoxide generation, but is also due to the activity of eNOS, when uncoupled. This uncoupling is both cause and consequence of the excess of peroxynitrite, which exhausts tetrahydrobiopterin, an essential cofactor for the proper function of eNOS. PON1 is deteriorated by other mediators, such as hypochlorite (more strictly, the pair hypochlorite/hypochlorous acid) and MPO, an enzyme closely related to the synthesis of such anions [41].

### 4.2. HDL Changes in Disease

There a general consensus in recognizing the value of wholly functional HDLc as anti-atherogenic, but also its detrimental effects on the vascular endothelium when HDL particles are altered in an oxidative and inflammatory milieu. Under these circumstances, HDL are damaged by the formation of MDA-protein adducts, a rather nonspecific transformation that causes a reduction of eNOS activity, as seen in patients of coronary disease. The detrimental consequences that such an event has on vascular function are counteracted by an increase in the activity of PON1 [28]. However, an inhibition of eNOS does not necessarily mean an inhibition of NO production since the iNOS pathway may be stimulated [10].

It has been reported that HDL isolated from patients with inflammatory conditions, such as diabetes, antiphospholipid syndrome, chronic kidney disease, and acute coronary syndromes, loses its capacity to directly stimulate endothelial NO production and impairs NO bioavailability, increases superoxide production, enhances adhesion molecule expression, and decreases paraoxonase activity in cultured endothelial cells [42,43,44]. Interestingly, one of the major findings from O’Neill et al. [45] was that acute changes in HDL function can recover with resolution of inflammation and are paralleled by changes in the HDL proteome. Furthermore, Sang et al. showed that the walk/run training in subjects with metabolic syndrome without a restricted diet leads to a significant improvement in HDL anti-inflammatory ability. The protective effect of HDL was tested by observing an increased eNOS expression and NO production in the presence of the subtype HDL3 isolated from those patients with injured endothelial cells after stimulation with tumor necrosis factor (TNF)-α. They suggested HDL-associated PON1 activity may be involved at least partially in the eNOS-derived NO production, as the activity of PON1 increased in both serum and HDL3 fractions from metabolic syndrome patients after 10 weeks of training. All these findings support the importance of HDL-associated PON1 in defining HDL vasodilatation properties through NO production [46].

Although most data indicate, as previously stated, that PON1 cooperates in the control of hypertension by means of increased eNOS activity, there is evidence that, via an unrelated mechanism, PON1 can promote high blood pressure. Gamliel-Lazarovich et al. reported that PON1-knockout mice showed lower systolic and diastolic blood pressure than that of their wild type congeners, and the difference was even more pronounced when the animals were treated with a high-salt diet. This result was interpreted in the light of the hydrolysis of the vasodilator 5,6-dihydroxy-eicosatrienoic lactone (5,6-DHTL). This reaction, catalyzed by PON1, gives 5,6-dihydroxy-eicosatrienoic acid, thus diminishing the renal content of both 5,6-DHTL and its precursor 5,6-epoxy-eicosatrienoic acid. The authors stressed the concordance with the fact that the 192RR genotype is both prone to hypertension and of an higher effectiveness in hydrolyzing lipid lactones [47]. Later, deeper analyses clearly demonstrated that 5,6-DHTL is in fact an intracellular substrate for the lactonase activity of PON1 [48]. The consequent loss of the lactone-cycle integrity impairs the increase of intracellular Ca^2+^, which is not only necessary for the K^+^ channel-dependent hyperpolarization of smooth muscle membrane, but also favorable for the activity of PON1. So, PON1 may be considered as a control knob of the vascular tone regulated by the closing/opening of the lactone ring of 5,6-DHTL [49].

## 5. Vascular Effects of Gonadal Hormones

The hormonal regulation of eNOS and bioavailability of NO have been attributed to playing a major role in maintaining vascular health during natural hormonal transitions with aging, diabetes, and metabolic disorders. Estrogens are among the hormones that enhance NO production and vascular relaxation [50]. Nuedling et al. demonstrated that 17β-estradiol up-regulates the expression of both eNOS and iNOS in neonatal and adult cardiac myocytes [51]. 17β-Estradiol also increases eNOS expression and NO in cultured human coronary artery endothelial cells. Mechanistically, two major pathways account for the increased function of eNOS by estrogens: a) rapid signaling by membrane estrogen receptor (ER) through the PI3K/Akt pathway resulting in eNOS phosphorylation and increased eNOS activity; and b) longer term-genomically regulated increasing in eNOS mRNA and protein [50].

Interestingly, 17β-estradiol has also been shown to positively influence the activity of PON1. On one hand, it was reported that women taking oral contraceptives presented higher basal paraoxonase, salt-stimulated paraoxonase, and arylesterase activities [52]; on the other, surgical menopause is associated with lower serum PON1 activity [53]. Additionally, hormone supplementation of female mice with combinations of progesterone and 17β-estradiol induced a two-fold increase in serum paraoxonase activity [54]. Consistently, a male human hepatoma cell line and normal male and female rat hepatocytes responded to 17β-estradiol with a dose-dependent increase in PON1 activity up to a maximal two- to three-fold increase. The underlying mechanism was hypothesized to be through increasing the specific activity and/or the catalytic stability of PON1 since 17β-estradiol did not induce higher levels of PON1 mRNA or PON1 protein nor increase PON1 secretion. In addition, it was also found that ApoA-I mRNA was not induced by 17β-estradiol, so the enhanced PON1 activity observed was likely not due to increased expression of ApoA-I [55].

Castardo-de-Paula et al. have recently shown that 17β-estradiol restores the PON1 enzyme activity in ovariectomized rats under constitutive NOS inhibition with *N*-ω-nitro-L-arginine methyl ester (L-NAME), an agent that causes long-term vascular oxidative effects in experimental models. Ovariectomy promotes a reduction in the activity of NOS and a pro-oxidant state, which may lead to reduced plasma PON1 activity, but those were prevented by treatment with 17β-estradiol. Provided that the L-NAME-mediated increase of mean arterial pressure was slight, the authors concluded that vasodilator status would arise from eNOS, whose activity is probably implicated in maintaining PON1 activity in ovariectomized rats. On the other hand, it was suggested that the higher increase in total plasma levels of NO in 17β-estradiol-treated rats was associated to iNOS activity [56].

Among steroid hormones, androgens have also been reported to be involved in eNOS activation through the PI3K/Akt signaling pathway. Although most of the biological actions of testosterone are mediated by androgen receptors (AR), some of them may be mediated by ER after conversion to 17β-estradiol [57]. Polycystic ovary syndrome (PCOS) is a common endocrine disease characterized by excess androgen levels and is considered a metabolic disorder associated with long-term health risks, since endothelial dysfunction is an important pathological component of PCOS [58]. Bayram et al. have measured significantly lower NO levels in PCOS patients as compared with that of normal healthy individuals. In addition, their findings indicate that hyperandrogenemia has a role in the formation of oxidative stress in PCOS, which was shown by higher serum MDA levels and lower PON1 activity [59]. Consistently, serum PON1 activity has been reported to be lower in male mice [60]. Bayrak et al. found that PON1-HTLase was significantly lower in a PCOS group. In turn, the lower PON1-HTLase activity was negatively correlated to asymmetric dimethylarginine (ADMA) levels, which were significantly higher in the PCOS group [61].

## 6. PON1 and Cholesterol Lowering Drugs

Statins are inhibitors of 3-hydroxy-3-methyl-glutaryl CoA reductase and are widely used drugs for hypercholesterolemia treatment and have cardioprotective and antioxidant effects regardless of their lipid-lowering effects. Some clinical and experimental studies have shown that atorvastatin increases PON1 activity [62,63], whereas some others failed to find any changes in serum paraoxonase activity following simvastatin or atorvastatin administration [64]. Some experiments with laboratory animals allowed a mechanistic approximation to that topic. For example, in the study by Sozer et al., the treatment of male Wistar rats with L-NAME resulted in both higher levels of oxidative stress and depression of antioxidant capacity. The administration of atorvastatin to L-NAME-treated rats decreased LDL-oxidation and serum concentrations of total cholesterol and LDLc, but it had no effect on the lower HDL-cholesterol concentrations induced by L-NAME. Interestingly, despite the low HDL-cholesterol concentrations, they found a significant increase in the PON1 activity in the L-NAME plus atorvastatin-treated group when compared with that of the L-NAME-treated rats. Therefore, it was suggested that the anti-atherosclerotic effect of atorvastatin shown in several studies seems to be associated to the effects of PON1, which impairs LDLc oxidation, besides its other pleiotropic effects [25]. In any case, extracting a clinically applicable conclusion is highly speculative, and the often contradictory results on PON1 activity exerted by statins reported in different studies could be explained by the existence of gene polymorphisms among individuals. These would make some patients have higher PON1 levels so they might obtain further benefit from the treatment [65,66].

Similar controversy exists with the improvement of the endothelial functions of hypercholesterolemic patients in treatment with statins. The protective effect of statins on endothelial function involves increasing NO biosynthesis and bioavailability via the direct up-regulation of eNOS expression [67]. In contrast to the above-mentioned study of Çiftci et al. [65], where atorvastatin behaved as a beneficial pharmacological modulator of impaired endothelial functions by decreasing ADMA levels, some other studies to date indicated that statins have no or little effect on ADMA levels [68,69].

Apart from statins, the long-term consumption of a traditional Mediterranean diet (TMD), enriched with virgin olive oil (TMD-VOO) or nuts (TMD-nuts), has recently shown to be able to increase the overall antioxidant protection of HDL on LDLs as well as increase PON1 arylesterase activity. In parallel, the TMD-VOO intervention increased the capacity of isolated HDL to induce the production of NO from endothelial cells. It was suggested that the high content of bioactive compounds in the TMD, especially when enriched with virgin olive oil, may act synergistically and could protect PON1 against oxidative modifications as well as enhance HDL function by increasing four key HDL functions: cholesterol efflux capacity, HDL-cholesterol metabolism, HDL antioxidant/anti-inflammatory properties, and vasoprotective effects [70].

## 7. The Special Case of a Classical PON Substrate: Homocysteine Thiolactone

Homocysteine (hCys) is a sulfur-containing non-protein amino acid derived from dietary methionine. Its accumulation is reflected in hyperhomocysteinemia, which is an analytical condition that has been associated with various cardiovascular, renal, and cerebral diseases [71]. The implication of PON1 in the deleterious vascular effects of hCys needs, perhaps, permanent clarification. On the one hand, this enzyme hydrolyzes homocysteine thiolactone (HTL), a form that in spite of accounting for only 1% of the total hCys in plasma, is considered the most dangerous form of the amino acid. However, it seems that the clinical relevance of HTL is smaller than initially assumed. In any case, for the purpose of the present review, it is noticeable that an excess of hCys participates in the process of post-transcriptional modification of proteins and causes oxidative stress, which in turn reduces eNOS and PON1 activities through the accumulation of MDA, as explained above. Particularly, some studies have implicated hCys as a risk factor for vascular disease, partially due to its instigation of vascular remodeling, which implies alterations in the content of the extracellular matrix and is regulated by NO generation [72]. There is conclusive evidence that high hCys levels significantly reduce NO synthesis and endothelial-dependent dilatation in response to a vasoactive stimulus, although the underlying mechanisms remain unknown.

Hyperhomocysteinemia has been shown to down-regulate the expression of Akt and eNOS in a study with endothelial progenitor cells [73]. The absence of Akt results in reduced eNOS phosphorylation, NO release, and endothelial cell migration [74]. HTL is the most reactive form generated from defective hCys metabolism and is responsible for hyperhomocysteinemia-induced vascular and tissue diseases. It binds with protein lysine residues and generates *N*-hCys-Lys with auto immunogenic and prothrombotic properties. HTL can be disposed of by enzymatic hydrolysis by the serum HTL-thioesterase (PON1) carried on HDL, thus leading to a negative correlation between plasma HTL-thioesterase and paraoxonase activities of PON1. Consistently, PON1 has been shown to protect proteins against *N*-homocysteinylation by hydrolyzing HTL [75]. Unfortunately, enzymatic activities of PON1 are diminished in pathological states. In vitro studies have reported that homocysteinylation of HDL may reduce the activity of the enzyme PON1, thus rendering the HDL particle more susceptible to oxidative damage [76]. In close relationship with the function of methyl-transference cofactors, Weijun et al. reported that short-term oral folic acid (5 mg/day) supplementation with or without methylcobalamin appeared to be an effective approach to decrease hCys levels and increase HTL-thioesterase/paraoxonase activity in patients with type 2 diabetes, which could be a novel mechanism to protect against vascular diabetic complications [77].

Givvimani et al. verified that the expression of eNOS and its activating kinase Akt were down-regulated in PON1-/- mice fed an atherogenic diet, while plasma hCys levels were increased. They found that expression of asymmetric dimethylarginine (ADMA), a potent endogenous competitive inhibitor of eNOS, was also significantly increased. Moreover, the expression of dimethylarginine dimethylaminohydrolase (DDAH), which is responsible for the metabolism of ADMA, was decreased in those mice [34]. Although previous studies had already reported that hyperhomocysteinemia mediates vascular dysfunction by increasing ADMA and down-regulating DDAH2 [78], the implication of PON1 needed to be explained. According to the authors, the lowering of the PON1 activity caused by hyperhomocysteinemia, or complete lack of PON1, as in knockout mice, makes the HDL dysfunctional, which leads to an imbalance in the ADMA and DDAH, resulting in decreased eNOS production [34,79]. In addition, hCys may contribute to enhanced oxidative inactivation of NO by its redox activity, which arises from the formation of disulfides and the generation of hydrogen peroxide and superoxide anions [69]. In turn, the oxidative stress and inhibition of NO release induced by hCys are suggested to promote a lower expression and activity of HDL-associated PON1 and enhance production of ROS in patients with hyperhomcysteinemia, leading to impaired antioxidant function and decreased capacity to degrade HTL [5,80,81].

## 8. NO and Its Oxidized Metabolites as Inhibitors of PON1-Driven Effects

The most direct interaction between NO and PON1 consists of the *S*-nitrosylation of the enzyme at the residue Cys284, thereby diminishing its enzymatic activity. This cysteine is also the target of inactivation by lipid peroxides. Kathib et al. studied recombinant human PON1 with different synthetic and physiological NO donors, and concluded that the most effective ones were *S*-NO-glutathione and serum *S*-NO-albumin. Serum albumin, which is nitrosylated by NO at its Cys34 residue in domain I, acts as a kind of store and mediator of the nitrosylating pathway that begins with by the genesis of NO by eNOS [82]. Perhaps each of the other relationships characterized by a detrimental effect of reactive nitrogen species on PON1 activity depend not on the effect of NO itself, but rather on its oxidized forms (NOx).

In this last section, a series of interactions that stem from the topics concerning HDL are considered. There are certain pathological entities, some of them studied in animal models and others in human beings, in which the increase of NO, often measured in terms of NOx, is accompanied by a decrease of PON1 activity. Referring to the animal experiments, the hepatic toxicity induced by tienilic acid in rats is marked by a reduction in PON1 activity, among many other indicators. It is noteworthy that PON1 inhibition is associated with increased inflammation, lipid peroxidation, and nitrative stress, measured by TNF-α, MDA, and 3-nitrotyrosine, respectively, to list just a few examples of its effects. An explanation of these observations could be given on the basis of the inactivation of cysteine thiol groups of PON1 by electrophylic metabolites of tienilic acid, thus enhancing the production of TNF-α, as seen in different models of pro-inflammatory oxidative stress. Furthermore, TNF-α amplifies the toxicity of tienilic acid, since it down-regulates PON1, and favors the genesis of additional amounts of NOx, through iNOS/NO [83].

In terms of clinical studies, the paper by Ames et al. on hepatopulmonar syndrome (HPS) and liver cirrhosis reveals that low PON1 levels are associated with higher nitrative effects. Their results express dramatic differences between HPS and control patients in serum PON1 activity, which strongly and inversely parallel those of NOx species. In every case, the analytical biochemical parameters were less altered than those measured in HPS with respect to the control values [84]. In rheumatoid arthritis, an inflammatory disease in which there is an abnormal autoimmune response to oxidation of the basic amino acids in proteins, a high amount of antibodies against HDL has been found, and they are credited to PON1. The consequent inactivation of PON1 is accompanied, as in HPS, by an increase in NOx levels, especially in the genotype 192QQ [85].

Maes et al. published a study that while undoubtedly narrow with reference to its demographic scope, is of high value for the deep analysis of nitrative stress and antioxidant defenses in patients suffering from mental generalized anxiety disease (GAD). This study took into account personal factors such as sex, age, body mass index, smoking habit, education, marital status, etc., along with central nervous comorbidities and their pharmacological treatments. Among the antioxidant physiological equipment, PON1 is one of the most important factors because its decrease is strongly associated to the presence of GAD. The authors reported the statistical significance of the incidence of 16 biochemical parameters including NOx, lipid peroxidation products, catalase, and protein carbonylation [86].

## 9. Concluding Remarks

The main conclusive judgment is that the presence of PON1 in plasma, isolated or co-ligated to other proteins in HDL, decisively favors the activity of eNOS and, therefore, the production of NO, vasodilators, and platelet anti-aggregates. It should be noted that this is only true in physiological conditions, because in a situation of oxidative stress, very common in diseases of all kinds, the presence of proinflammatory cytokines and the final products of lipid peroxidation, essentially MDA, deprive PON1 and HDL of their vascular-protective characters. In some cases, it has been seen that PON1 increases blood pressure by a mechanism independent of NO, due to the hydrolysis of vasodilator lipid lactones. The iNOS activity, which by its nature leads to an abrupt production of higher amounts of NO, when coinciding with other stimuli, causes NO to be transformed into peroxynitrite, which due to the tyrosine-nitrative and lipid pro-oxidant effects of peroxynitrite, and might eventually lead to structural alterations of PON1 and thereby to a modification of PON1’s activity. Finally, it should be mentioned that NO, as such, can exert an inactivating effect on PON1 by a post-translational reaction of *S*-nitrosylation of cysteine.
